# Metabolic Dependencies Underlie Interaction Patterns of Gut Microbiota During Enteropathogenesis

**DOI:** 10.3389/fmicb.2019.01205

**Published:** 2019-06-04

**Authors:** Die Dai, Teng Wang, Sicheng Wu, Na L. Gao, Wei-Hua Chen

**Affiliations:** ^1^Key Laboratory of Molecular Biophysics of the Ministry of Education, Hubei Key Laboratory of Bioinformatics and Molecular-Imaging, Department of Bioinformatics and Systems Biology, College of Life Science and Technology, Huazhong University of Science and Technology, Wuhan, China; ^2^College of Life Science, Henan Normal University, Xinxiang, China; ^3^Huazhong University of Science and Technology Ezhou Industrial Technology Research Institute, Ezhou, China

**Keywords:** bacterial interaction patterns, metabolic interaction network, gut microbiota community, intestinal microbial ecology, enteropathogenesis, probiotics

## Abstract

In recent decades, increasing evidence has strongly suggested that gut microbiota play an important role in many intestinal diseases including inflammatory bowel disease (IBD) and colorectal cancer (CRC). The composition of gut microbiota is thought to be largely shaped by interspecies competition for available resources and also by cooperative interactions. However, to what extent the changes could be attributed to external factors such as diet of choice and internal factors including mutual relationships among gut microbiota, respectively, are yet to be elucidated. Due to the advances of high-throughput sequencing technologies, flood of (meta)-genome sequence information and high-throughput biological data are available for gut microbiota and their association with intestinal diseases, making it easier to gain understanding of microbial physiology at the systems level. In addition, the newly developed genome-scale metabolic models that cover significant proportion of known gut microbes enable researchers to analyze and simulate the system-level metabolic response in response to different stimuli in the gut, providing deeper biological insights. Using metabolic interaction network based on pair-wise metabolic dependencies, we found the same interaction pattern in two IBD datasets and one CRC datasets. We report here for the first time that the growth of significantly enriched bacteria in IBD and CRC patients could be boosted by other bacteria including other significantly increased ones. Conversely, the growth of probiotics could be strongly inhibited from other species, including other probiotics. Therefore, it is very important to take the mutual interaction of probiotics into consideration when developing probiotics or “microbial based therapies.” Together, our metabolic interaction network analysis can predict majority of the changes in terms of the changed directions in the gut microbiota during enteropathogenesis. Our results thus revealed unappreciated interaction patterns between species could underlie alterations in gut microbiota during enteropathogenesis, and between probiotics and other microbes. Our methods provided a new framework for studying interactions in gut microbiome and their roles in health and disease.

## Introduction

In recent decades, increasing evidence has strongly suggested that gut bacteria play an important role in human health and disease (Selber-Hnatiw et al., 2017; Jackson et al., 2018). Gut bacteria has been considered as a real tissue with its specific functions such as modulating the metabolic phenotype, influencing innate immunity, protecting against pathogens, and so on (Eckburg et al., 2005; Tomasello et al., 2017). Changes in the composition of the gut microbiota have been proven to be associated with many diseases (Jackson et al., 2018) including inflammatory bowel disease (IBD; Joossens et al., 2011; Matsuoka and Kanai, 2015; Chu et al., 2016; Sartor and Wu, 2017; Zuo and Ng, 2018), type 2 diabetes (Delzenne et al., 2015), obesity (Moreno-Indias et al., 2014; Tai et al., 2015), atherosclerosis (Drosos et al., 2015; Yamashita et al., 2015) and colorectal cancer (CRC; Aarnoutse et al., 2017; Liang et al., 2017; Russo et al., 2018). Among which, IBD (Miyoshi and Chang, 2017; Sartor and Wu, 2017), including both Crohn’s Disease (CD) and ulcerative colitis (UC), is one of the most-studied imbalances between intestinal microflora and the immune system. Over the past 50 years, there was a dramatic increase in IBD (Sartor and Wu, 2017). In addition, patients with IBD are at increased risk of CRC, accounting for less than 2% of colon cancer cases yearly (Tilg et al., 2018). CRC, one of the most common cancers with the highest mortality worldwide, has also been reported to be associated with intestinal microflora (Zeller et al., 2014).

Gut microbes live as a community, sharing the common intestinal environment (Shetty et al., 2017). They interact with each other, maintaining the intestinal microbial flora in a state of equilibrium (Sommer et al., 2017). The composition of gut microbiota is thought to be largely shaped by interspecies competition for available resources along with cooperative interactions (Zelezniak et al., 2015). Diet is considered as one of the main drivers (De Filippo et al., 2010), with certain contributions from intrinsic metabolic dependencies. However, to what extent the changes could be attributed to external factors like diet of choice and internal factors such as mutual relationships among gut microbiota, respectively, are yet to be elucidated. Furthermore, it is still unclear how such intrinsic dependencies could contribute to the parthenogenesis of intestinal diseases such as IBD and CRC.

In this study, we performed systematic network analysis based on pairwise interspecies metabolic dependencies among gut microbes in IBD and CRC patients and compared that of the healthy controls. Network analysis has proven to be a valuable tool in exploring interactions between a set of items (nodes, such as individuals in a school, species in a complex food web, proteins in metabolic pathways) by biologists and scientists in other fields (Kim and Hastak, 2018), and has recently been applied to explore and identify microbial patterns that are generally difficult to detect in complex systems (Chow et al., 2014; Cardinale et al., 2015; Kong et al., 2018). Due to the advances of high-throughput sequencing technologies, flood of (meta)-genome sequence information and high-throughput biological data are available for gut microbiota and their association with intestinal diseases, making it easier to gain understanding of microbial physiology at the systems level (Covert et al., 2004). In addition, the newly developed genome-scale metabolic models that cover significant proportion of known gut microbes enable researchers to analyze and simulate the system-level metabolic response in response to different stimuli in the gut, providing deeper biological insights (Zhang and Hua, 2016; Magnusdottir et al., 2017; van der Ark et al., 2017). Based on these data, we revealed unappreciated patterns in gut microbes of IBD and CRC patients and healthy controls, and were able to accurately predict the majority of the changes (i.e., decreased or increased) in the gut microbiota during enteropathogenesis. As compared with co-occurrence network (Cardinale et al., 2015), which has been widely applied in the identification and characterization of interspecies interactions among gut microbes, our metabolic dependency network is a directional network and can provide more information with considering the interaction between the bacteria. We thus concluded that metabolic dependencies underlie interaction patterns of gut microbiota community during enteropathogenesis, and believed that our methods could provide a new framework for studying interactions in gut microbiome and their roles in health and disease.

## Materials and Methods

### Data Collection

#### Pair-Wise Interactions (Metabolic Dependencies) of Human Gut Microbes

Genome-wide metabolic models for 773 human gut microbes were obtained from Stefanía et al. (Magnusdottir et al., 2017). Pairwise interactions, i.e., changes *in silico* growth rates of two co-culturing microbes as compared with that of cultured alone were calculated using the methods described in the literature (Magnusdottir et al., 2017).

Briefly, genome-scale metabolic models of 773 human gut microbes described in literature (Magnusdottir et al., 2017) were reconstructed based on comparative genomics and enrichment literature-derived experimental data. Through a combination of detailed biochemical information from genome annotations and literature resources, genome-scale metabolic models can be constructed. The gene-protein-reaction (GPR) relationships are annotated in the metabolic modes with mass- and energy-balanced reactions. Furthermore, other omics data such as transcriptomic and proteomic data could be integrated into the model, making the model more informative. Additionally, pairwise simulations were performed on every pair of 773 microbes (298,378 pairs). Single and pairwise *in silico* growth rates were calculated on two different diets (Western and High fiber diet).

Based on these growth rates, we calculated the “weight” of the interaction between bacteria using the following equation, $w\;=\;{\text{Log}}_{2}\frac{P}{S}$, where *P* stands for growth rate of the species of interest when co-cultivated with another bacterium (paired growth rate) and *S* stands for growth rate when cultivated alone. A “*w*” value of 0 indicates the growth rate of a bacterium is not changed by the other co-cultivated bacterium; a positive (negative) value of “*w*” indicates the growth rate can be promoted (inhibited) by the co-cultivated bacterium. The interactions between two bacteria are thus bi-directional.

#### Gut Metagenomic Data of IBD and CRC Patients and Healthy Controls

In total three metagenomic datasets, including two for IBD and one for CRC, were obtained from the European Nucleotide Archive (ENA; Leinonen et al., 2011)^1^ database.

The first IBD datasets (referred to as IBD1 in our study) are available from ENA under the accession of ERP005534. It contained ten IBD and ten healthy individuals whose fecal microbiome compositions were determined using Illumina HiSeq 2500.

The second IBD datasets [ENA accession ID: SRP002423; referred to as IBD2 (NIH HMP Working Group et al., 2009; Noecker et al., 2016) in our study] contained 14 healthy samples and 20 disease samples; their fecal samples were sequenced using a 454 GS FLX Titanium pyrosequencer. This study is a part of the NIH Human Microbiome Project (HMP).

The third CRC datasets [ENA accession ID: ERP005534; referred to as CRC (Zeller et al., 2014) in our study] contained fecal samples of 53 patients and 61 healthy controls. In this study, metagenomic sequencing of fecal samples was used to identify potential markers for distinguishing CRC patients from tumor-free controls. The detailed description about the experiments actually entailed can be found in the literature (Zeller et al., 2014). In brief, fresh stool samples were collected and genomic DNA was extracted using the GNOME DNA Isolation Kit (MP Biomedicals). Then library preparation for metagenomic sequencing was automated and adapted on a Biomek FXp Dual Hybrid. And metagenomic sequencing was performed on the Illumina HiSeq 2000/2500 platform.

### Read Processing and Quality Control

Trimmomatic (Bolger et al., 2014) was used to remove adaptors and low quality bases (trimming) from the Illumina paired-end and single-end reads. For Roche/454 sequence data, QTrim (Shrestha et al., 2014) was used for trimming. FastQC (Andrews, 2014)^2^ was then used for quality control prior to downstream analysis; the generated HTML report files were manually examined for possible problems in the raw and processed data. The usable trimmed data were referred to as “Clean Data,” and were used for downstream analysis.

### Species Identification and Composition Analysis of Metagenomic Data

MetaPhlAn2 (Metagenomic phylogenetic analysis version 2; Truong et al., 2015) was used for the taxonomic composition analysis on the Clean Data with default parameters. MetaPhlAn2 can efficiently profile the composition of microbial communities with species level resolution.

### Differential Abundance Analysis Between Disease and Healthy Samples

Wilcoxon Rank Sum test was used to identify differentially abundant species between patients and healthy controls. The detailed results are available in Supplementary Table S1. Supplementary Figure S1 shown in is the boxplot of the relative abundances of identified species in IBD1 patients (red) and healthy controls (blue); the red (blue) dots under the box plots represent a significant decrease (increase) in the abundance in disease group. The classification of the bacteria (Commensal, Pathogen, and Probiotic) is provided by the literature (Magnusdottir et al., 2017), which is shown in Supplementary Table S2.

### Construction and Characterization of Metabolic Dependency Network for Disease and Healthy Controls

The metabolic dependency networks were constructed using pairwise interactions and consisted of nodes and edges. Networks were constructed for each of the three datasets we collected, and separately for patients and healthy controls. For each network, the nodes were microbial species selected from the union of the top 50 most abundance species in patients and the respective healthy controls, whose combined account for more than 90% of the total abundances of all species, while the edges were pairwise interactions (“weights”) between two connected species. To account for the impact of diets [Western and High fiber diet, as described in the literature (Magnusdottir et al., 2017)], two networks were constructed for each of the patient and control groups. At the end, four networks were obtained for each dataset. An open-source tool, Gephi (Bastian et al., 2009), was used for network visualization and analysis.

### Statistics

All statistical analysis and plots were performed in R version 3.4.3^3^. Mann–Whitney and Chi square test were used to analyze differences between groups. The *p*-value < 0.05 was considered significant.

## Results

### Construction of Metabolic Dependency Network for Gut Microbiota During Enteropathogenesis

The flow chart of the methods used is shown in Supplementary Figure S2. We collected gut metagenomics data from in total three published datasets, including two for IBD (NIH HMP Working Group et al., 2009; Noecker et al., 2016) and one for CRC (Zeller et al., 2014), each with different numbers of patients and healthy controls (see section “Materials and Methods” for details). We first constructed a metabolic dependency network for each of the sample groups (i.e., patients and controls). Briefly, MetaPhlAn2 was used for the taxonomic composition analysis on the clean data with default parameters. The nodes in the network are microbial species selected from the union of the top 50 most abundance species that together account for more than 90% of the total abundances of all species in healthy and disease groups, while the edges represent pairwise interactions between two connecting species. The weight of the edge is the absolute value of the influence, which equals to log2-transformed growth rates change between co-culturing and single-growth (i.e., the growth rate when cultivated alone). The edges are thus directional; depending on the thresholds of the weights of the edges, there could be two edges connecting two neighboring nodes in the network, with each representing the impact of co-culturing as compared with the respective single-growth. Because the growth rate under co-cultured conditions could be slower than that of the single-culturing, we used red (green) to represent the increased (decreased) growth rate under co-cultured conditions.

For each of the three studies from where our data were obtained, we constructed networks for the patients and the respective healthy controls separately. To account for the impact of diet [western diet and a high fiber diet, as described in Magnusdottir et al. (2017)], we constructed two networks each of the patient and control groups. At the end, we obtained four networks for each dataset. In this study, we described the results of IBD1 in western diet as an example, other datasets produced approximately the same results which were not shown here.

### Network Centrality Analysis Revealed Probiotics Are Among the Top Important Nodes

**Figure 1:**
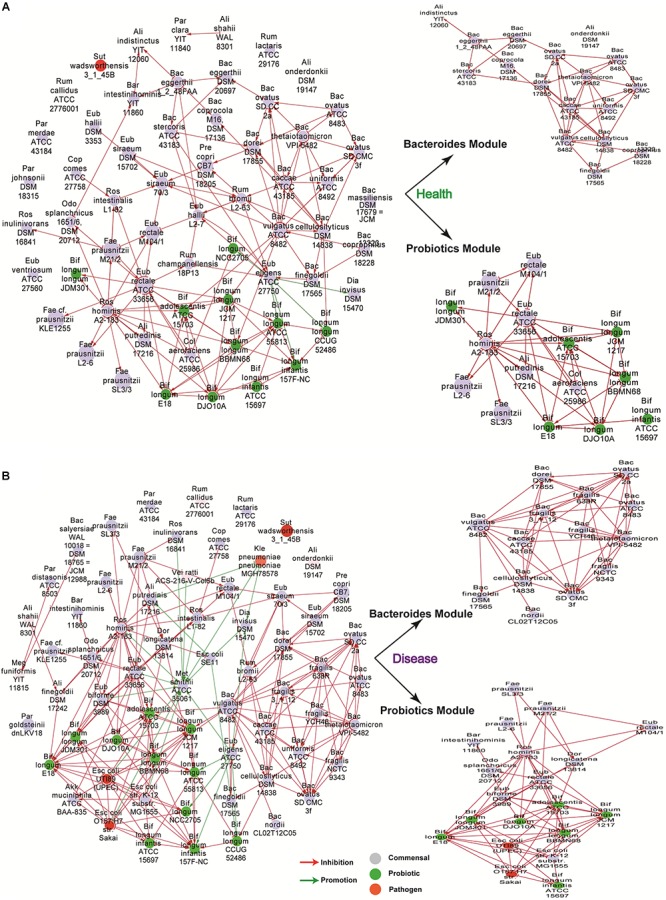
The bacteria interaction networks (weight > 4) obtained from healthy controls **(A)** and patients **(B)**. ForceAtlas2 layout in Gephi (Bastian et al., 2009) was used for this representation. Nodes filled with gray, green, red, and pink represent commensal, probiotic, pathogenic, and opportunistic pathogenic bacteria, respectively. Two main subclusters were identified, one includes mostly probiotic bacteria (Probiotics Module), while the other consists mostly of species in the genus bacteroides (Bacteroides Module).

As shown in Figure 1, we included dependencies of weight greater than four to build the network for healthy and disease groups respectively. We used gray, green, red, and pink to indicate nodes for commensal, probiotic, pathogenic, and opportunistic pathogenic bacteria, respectively, using classifications from a public dataset (Magnusdottir et al., 2017). To identify subclusters in which nodes are more densely connected than to the rest of the network, we used a modularity algorithm (a “community” detection technique) implemented in Gephi (Bastian et al., 2009) and identified two main subclusters (Figure 1); among which, one was mainly composed of probiotics bacteria, while the other was mostly composed of species of the genus of bacteroides. Surprisingly, we found that some pathogen bacteria, such as some strains of Escherichia coli, was also included in the probiotics subcluster and had a notable inhibitory effect on the probiotics included (Figure 1).

**Figure 2:**
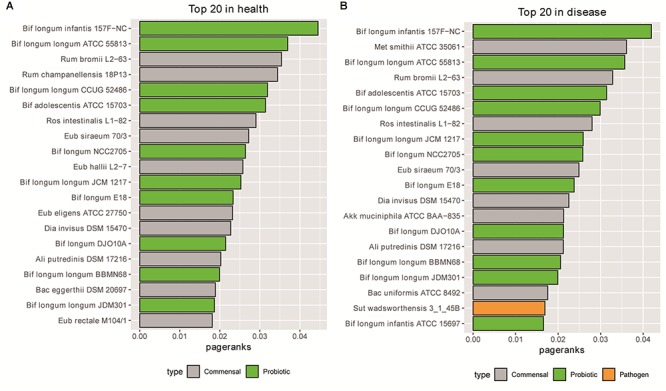
Most of the top 20 bacteria based on PageRanks are probiotics in health **(A)** and disease **(B)** states.

We then checked the top important nodes in the metabolic dependency network. We used the Gephi’s PageRank algorithm (Chen et al., 2007) to rank the nodes. In addition to network centrality, PageRank also considers both the inbound and outbound links, which is suitable for analyzing our metabolic dependency network. Strikingly, we found that most of the top 20 bacteria were probiotics (10/11 in health and 11/11 in disease states), as shown in Supplementary Tables S3, S4 and Figure 2. These results thus indicate that probiotics may play important roles in the metabolic dependency network.

### Growth of Probiotics Was Strongly Inhibited by Other Bacteria in Both Patients and Healthy Controls

**Figure 3:**
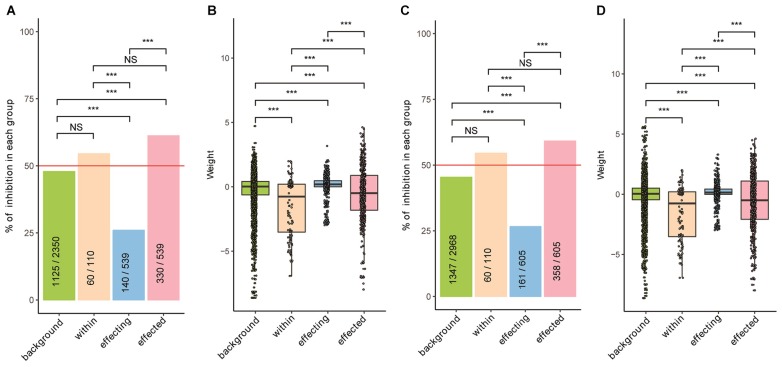
The growth of probiotics was strongly inhibited by other bacteria in both patients and healthy controls. **(A,C)** Proportions of inhibitory interactions in the four groups, calculated separately for patients **(A)** and healthy controls **(C)**; Chi-square test was used to test pairwise differences between two groups. **(B,D)** Distribution of weight values in the four groups, calculated separately for patients **(B)** and healthy controls **(D)**; Wilcoxon Rank Sum test was used for pairwise comparisons between two groups. Interaction data of the four groups are: background – interactions among bacteria excluding probiotics; within – interactions among probiotics; affecting – impacts of probiotics on others; affected – impacts of others on probiotics. Level of significance: NS – not significant; ^∗∗∗^*p* < 0.01.

Strikingly, we found that the growth of probiotics topped the centrality analysis was strongly inhibited by themselves and others; we found similar results in patients and healthy controls. As shown in Figure 3, we divided the interactions into four groups. First, the background group includes interactions among bacteria excluding the probiotics. Second, the within group includes interactions among probiotics. Third, the affecting group includes the impacts of probiotics to other bacteria. Fourth, the affected group includes the impacts of other bacteria on probiotics. We found that the weight scores were significantly lower in the “within” and “affected” groups as compared with the other two; we found similar trends in both patients and the controls (Figure 3B,D, respectively; Wilcoxon Rank Sum test). Similarly, we found that the proportion of inhibitory effects in the “affected” were significantly higher than other three groups (Figure 3A,C; Chi-square test). The “with” group contained significantly higher proportion of inhibitory effects than the “affecting” group; its proportion was also higher than that of the background, although the difference was not significant. These results indicate that although probiotics are mostly beneficial to the host, they often face competition from other probiotics and are clearly not welcomed by other.

### Disease-Enriched Bacteria Are Boosted by Themselves as Well as Other Bacteria

**Figure 4:**
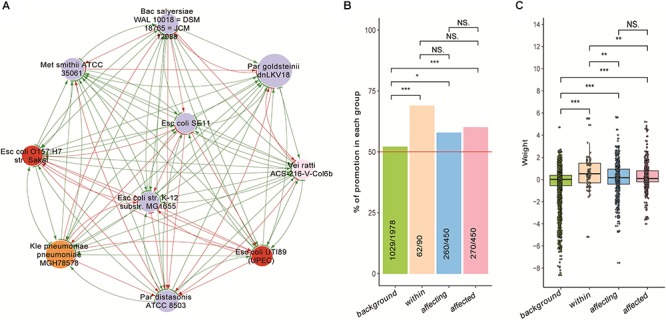
The growth of disease-enriched bacteria could be promoted by themselves and others. **(A)** An exemplary network of disease-enriched bacteria. **(B)** Proportion of promoting interactions in each group; chi-square test was used to perform pairwise (two-groups at a time) comparisons. **(C)** Distribution of weight values in the four groups; Wilcoxon Rank Sum test was used for pairwise comparisons. Interaction data of the four groups are: background – interactions among bacteria excluding probiotics and disease-enriched ones; within – interactions among disease-enriched bacteria; affecting – impacts of disease-enriched bacteria on others; affected – impacts of others on disease-enriched bacteria. Level of significance: NS – not significant; ^∗^*p* < 0.05; ^∗∗^0.01 < *p* < 0.05; ^∗∗∗^*p* < 0.01.

We found that the growth of bacteria whose abundances were significantly increased in patients (then were hence referred as to “disease-enriched bacteria”) could be promoted by themselves as well as by others. We divided pairwise interactions into four groups. First, the background group contains interactions excluding the disease-enriched bacteria and the probiotics. Second, the within-group includes interactions among the disease-enriched bacteria. Third, the affecting group includes the impacts of disease-enriched bacterial on others. Fourth, the affected group includes the impact of other bacteria on the disease-enriched ones. As shown in Figure 4, there were significantly more promoting affects in the second and third as compared with other two groups (Figure 4B,C), indicating a marked difference of the disease-enriched bacteria as a group as compared to others.

### Alterations of the Gut Microbiota During Enteropathogenesis Can Be Explained by Their Immediate Neighbors in the Metabolic Dependency Network

We next checked if alterations of the gut bacteria could be explained by their immediate neighbors in the network. For a given node (species) in the network, we considered two parameters in this calculation, namely the weight of the interactions (w) and the relative abundances (a) of its connecting nodes, and calculated an Inbound Influence Index using following equation: $\sum \left(\bar{w}\;\times \;a\right)$. As shown in Table 1 and Supplementary Table S5, we were able to predict up to 75% of the directions (i.e., increase or decrease) of the nodes in the metabolic dependency network.

**Table 1:** The recognition accuracy for the three datasets (analyzed in different diet).

	Accuracy (%)
IBD10_mphlan_HF	53.10
IBD10_mphlan_W	65.60
twin_mphlan_HF	72.70
twin_mphlan_W	63.60
CRC_mphlan_HF	57.14
CRC_ mphlan_W	47.62

## Discussion

In this study, we constructed metabolic dependency networks using gut microbiota datasets of common entero-diseases including IBD and CRC, and revealed unappreciated interaction patterns of disease-enriched bacteria and probiotics. In addition, we showed that the alterations of the gut microbiota during enteropathogenesis can be explained by their immediate neighbors in the metabolic dependency network with reasonable accuracy.

We used Wilcoxon Rank Sum test to identify differentially abundant species between patients and healthy controls. Although the identified significantly changed bacteria are quite different in the two IBD datasets (both contained patients and healthy controls, see Supplementary Table S1), we found similar interaction patterns (“mutual inhibition” between probiotics and “mutual promotion” between those significantly enriched bacteria) in the two IBD datasets and the CRC dataset.

Here, the classification of the bacteria (Commensal, Pathogen, and Probiotic) is provided by the literature (Magnusdottir et al., 2017), which is shown in Supplementary Table S2. Some strains in *Bifidobacterium bifidum*, which belong to the probiotics, were identified as the most variable strains between the healthy and disease. It is generally known that probiotics can improve human health. A precise definition of probiotics has been proposed by Laurent Verschuere (Verschuere et al., 2000). It was defined as a live microbial adjunct which has a beneficial effect on the host by modifying the host-associated or ambient microbial community, by enhancing the host response toward disease, by improving the quality of its ambient environment, or by ensuring improved use of the feed or enhancing its nutritional value. Above all, the most commonly purported benefits of the consumption of probiotics is modulation of host immunity (Corthésy et al., 2007). Because of these merits, the market for probiotics and probiotic-containing commercial products is constantly growing (Marco et al., 2006; Varankovich et al., 2015). However, a stable microbial community cannot be achieved by a sudden increase in nutrients due to exogenous feeding with probiotics (Verschuere et al., 2000). And we report here for the first time that there is a tendency of mutual restrain between the probiotic bacteria. Therefore, it is very important to take the mutual interaction of probiotics into consideration when develop probiotics or “microbial based therapies.”

With the growing recognition of the profound impacts of gut microbiota on human health, it is urgent to understand the molecular basis underlying the alterations of individual species in this complex microbial ecosystem. Compared to the undirected co-occurrence network, the metabolic dependency network is directional and thus could provide mechanistic insights into interspecies interactions. Numerous previous studies have suggested that host genetic and environmental factors can influence the diversity and composition of the gut microbiota (Benson et al., 2010). Among the environmental factors, dietary habits has proven to play a dominant role over other possible variables such as geography, climate, sanitation, hygiene, and ethnicity in shaping the gut microbiota (De Filippo et al., 2010; Walker et al., 2011). Our results indicate that at least in part, the alterations of the gut microbiota under different healthy statuses of the hosts, could be attributed to internal factors including species-species interactions of the gut microbes.

Using metabolic interaction network based on pair-wise metabolic dependencies, we found that unappreciated interaction patterns of between-species metabolic interactions could underlie alterations in gut microbiota during enteropathogenesis, and between probiotics and other microbes. Our methods provided a new framework for studying interactions in gut microbiome and their roles in health and disease. Though carefully evaluated, our results are still highly predictive and to be experimentally validated in the future.

## Author Contributions

W-HC and DD designed the study. TW and SW collected the data. DD analyzed the data and wrote the first draft of the manuscript. W-HC and NG contributed to the manuscript revisions. All authors approved the submission.

## Conflict of Interest Statement

The authors declare that the research was conducted in the absence of any commercial or financial relationships that could be construed as a potential conflict of interest.

## Footnotes

1 http://www.ebi.ac.uk/ena

2 http://www.bioinformatics.babraham.ac.uk/projects/fastqc/

3 www.r-project.org
