# Supplementary material for: Metabolic Dependencies Underlie Interaction Patterns of Gut Microbiota During Enteropathogenesis
Source: Front Microbiol. 2019 Jun 4;10:1205. doi: 10.3389/fmicb.2019.01205 (PMC6558107; doi:10.3389/fmicb.2019.01205)
Supplement: Supplementary file 6 [file Data_Sheet_1.docx]

**SUPPLEMENTAL MATERIAL**


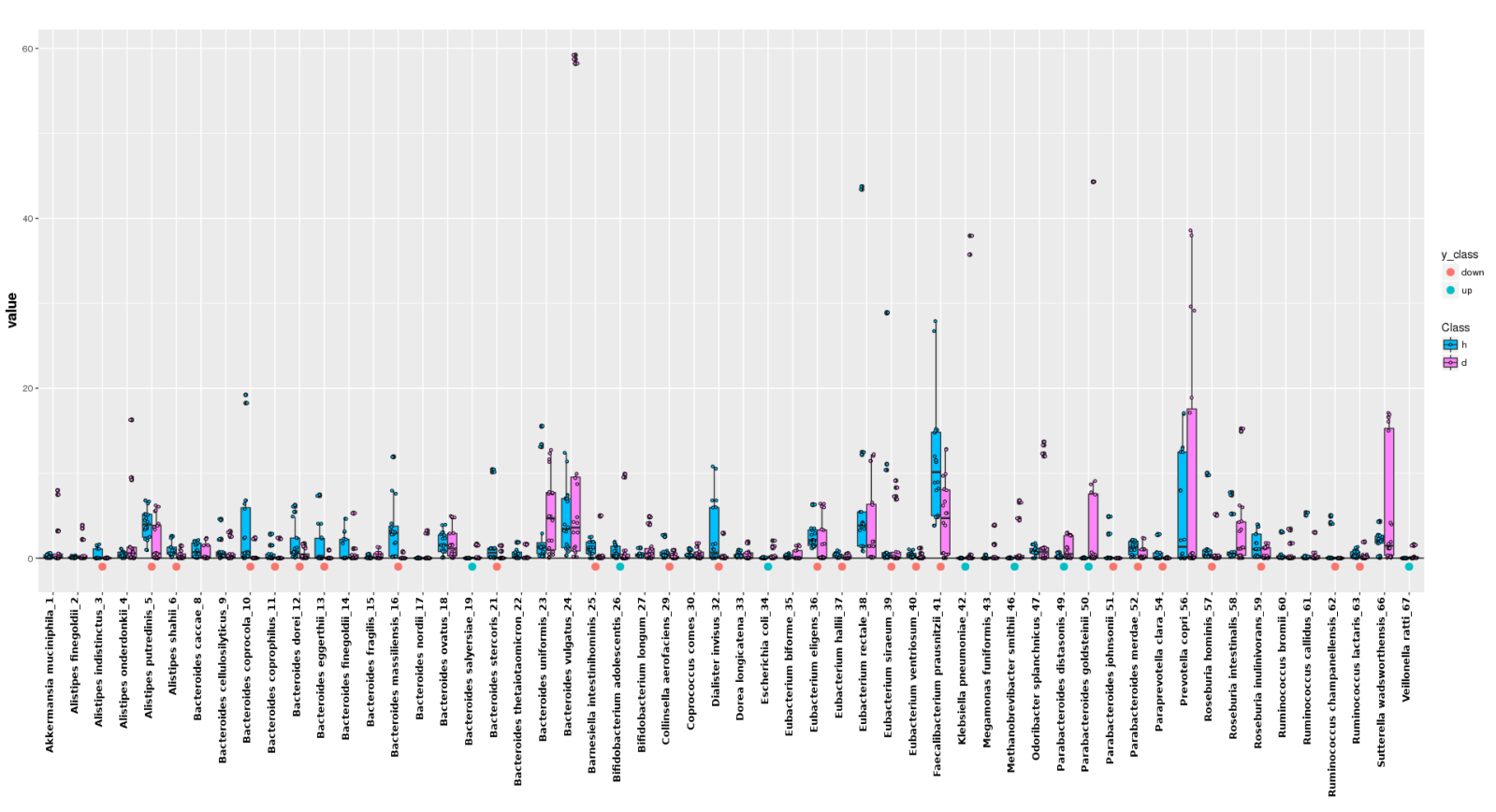


Figure S1 Boxplots of the relative abundance of bacteria in the dataset of IBD1 in western diet analyzed by metaphlan2. The blue box plot represents the distribution of the abundance in healthy group, and the red one represents the disease group. The red dots under the box plots represent a significant decrease in the abundance in disease group, while the blue ones represent a significant increase.


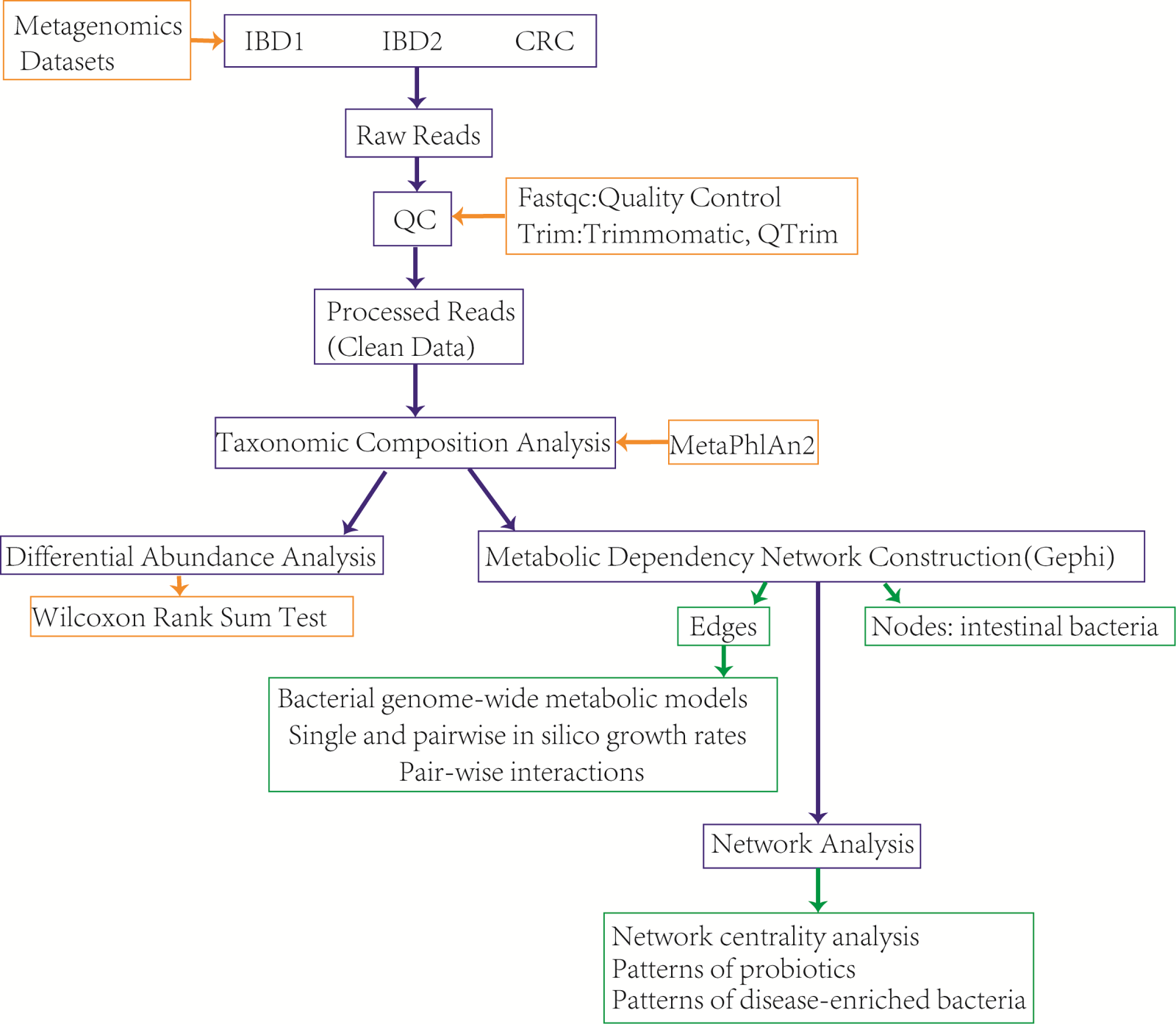


Figure S2 Flow chart of the methods used in the paper.
